# Identification of Active Retinaldehyde Dehydrogenase Isoforms in the Postnatal Human Eye

**DOI:** 10.1371/journal.pone.0122008

**Published:** 2015-03-20

**Authors:** Angelica R. Harper, Allan F. Wiechmann, Gennadiy Moiseyev, Jian-Xing Ma, Jody A. Summers

**Affiliations:** 1 Department of Cell Biology, University of Oklahoma Health Sciences Center, Oklahoma City, Oklahoma, United States of America; 2 Department of Physiology, University of Oklahoma Health Sciences Center, Oklahoma City, Oklahoma, United States of America; University of Florida, UNITED STATES

## Abstract

**Background/Objectives:**

Retinaldehyde dehydrogenase 2 (RALDH2) has been implicated in regulating all-*trans*-retinoic acid (atRA) synthesis in response to visual signals in animal models of myopia. To explore the potential role of retinaldehyde dehydrogenase (RALDH) enzymes and atRA in human postnatal ocular growth, RALDH activity, along with the distribution of RALDH1, RALDH2, and RALDH3 in the postnatal eye was determined.

**Methodology:**

Retina, retinal pigment epithelium (RPE), choroid, and sclera were isolated from donor human eyes. RALDH catalytic activity was measured in tissue homogenates using an *in vitro* atRA synthesis assay together with HPLC quantification of synthesized atRA. Homogenates were compared by western blotting for RALDH1, RALDH2, and RALDH3 protein. Immunohistochemistry was used to determine RALDH1 and RALDH2 localization in posterior fundal layers of the human eye.

**Principal Findings:**

In the postnatal human eye, RALDH catalytic activity was detected in the choroid (6.84 ± 1.20 pmol/hr/ug), RPE (5.46 ± 1.18 pmol/hr/ug), and retina (4.21 ± 1.55 pmol/hr/ug), indicating the presence of active RALDH enzymes in these tissues. RALDH2 was most abundant in the choroid and RPE, in moderate abundance in the retina, and in relatively low abundance in sclera. RALDH1 was most abundant in the choroid, in moderate abundance in the sclera, and substantially reduced in the retina and RPE. RALDH3 was undetectable in human ocular fundal tissues. In the choroid, RALDH1 and RALDH2 localized to slender cells in the stroma, some of which were closely associated with blood vessels.

**Conclusions/Significance:**

Results of this study demonstrated that: 1) Catalytically active RALDH is present in postnatal human retina, RPE, and choroid, 2) RALDH1 and RALDH2 isoforms are present in these ocular tissues, and 3) RALDH1 and RALDH2 are relatively abundant in the choroid and/or RPE. Taken together, these results suggest that RALDH1 and 2 may play a role in the regulation of postnatal ocular growth in humans through the synthesis of atRA.

## Introduction

Postnatal ocular growth is regulated by a vision-dependent process that coordinates the growth of the eye such that the ocular axial length will align the retina with the focal plane to give clear, uncorrected vision [1]. Interruption of this process can result in abnormal axial elongation of the eye, often leading to the development of myopia or nearsightedness due to altered scleral extracellular matrix remodeling at the posterior pole of the eye [2–4]. Studies using a variety of animal models have suggested that visually guided ocular growth is regulated locally (within the eye) via a retina-sclera chemical cascade [5].

All-*trans*-retinoic acid (atRA) has been implicated as a key signaling molecule in the regulation of postnatal eye growth in chicks, guinea pigs, and primates [6–10]. It is speculated that atRA regulates scleral matrix remodeling and ocular size through stimulation or repression of transcription factors, extracellular matrix constituents, matrix metalloproteinases (MMPs) and/or tissue inhibitors of MMPs (TIMPs) [7]. Therefore, atRA may represent a chemical signal that can directly modulate ocular size and refraction in many vertebrate species.

atRA is a small molecule metabolite of retinol, also known as vitamin A, that is required for the growth and development of many major organ systems in a variety of animal species. atRA is synthesized through a two-step process: 1) oxidation of retinol to retinaldehyde in a reversible reaction involving the retinol dehydrogenase (RDH) family of enzymes, and 2) oxidation of retinaldehyde to retinoic acid in an irreversible reaction catalyzed by one of three retinaldehyde dehydrogenase enzymes (RALDH1, RALDH2, RALDH3; also known as ALDH1α1, ALDH1α2, ALDH1α3, respectively). *In vivo*, endogenous atRA concentrations are spatially and temporally regulated to generate atRA concentration gradients, which have been shown to be necessary for morphogenesis and patterning of a variety of developing systems [11–13]. Using ultrasensitive liquid chromatography tandem mass spectrometry (LC/MS/MS), endogenous atRA concentrations in postnatal mouse tissues have been shown to range from 38.1 pmol/g in the liver to 1.5 pmol/g in muscle [14].

It is well established that the three RALDH enzymes cooperatively mediate retinoic acid signaling during eye development through tightly regulated spatiotemporal expression patterns [15–17]. The role of these enzymes in the postnatal eye is less well-characterized. In humans, RALDH1 constitutes 3% and 2% of the soluble proteins in the cornea and lens epithelium, respectively [18]. RALDH1 expression in the cornea and lens has been proposed to protect inner ocular tissues from ultraviolet radiation and reactive oxygen-induced damage [19]. Additionally, RALDH1 is considered a crystallin protein, present in the lens and cornea where it is responsible for the cellular transparency of these tissues [19].

Summers Rada *et al*. (2012) identified RALDH2 as the key enzyme responsible for increased synthesis of atRA in the chick choroid during periods of ocular growth deceleration (recovery from form deprivation induced myopia) [7]. In the postnatal chick eye, steady state RALDH2 mRNA levels were significantly increased in choroids following 12 hours of recovery from previously induced myopia and gradually returned to control levels following 15 days of recovery. Increased RALDH2 mRNA expression corresponded to increased endogenous concentrations of atRA in recovering choroids of approximately 1 x 10^−8^ M [7].

RALDH2 mRNA and protein were shown to be expressed by a unique subpopulation of extra-vascular choroidal stromal cells in the chick choroid, some of which co-expressed α-smooth muscle actin [7]. It was suggested that increased choroidal expression of RALDH2 during recovery leads to increased production of atRA which may act directly on the sclera to slow ocular elongation. Interestingly, RALDH2 protein expression was also shown to be increased in the retina of juvenile guinea pig eyes during lens-induced myopia [20]. Therefore, through its regulation of atRA synthesis, RALDH2 may represent a visually regulated gene that has a direct effect on scleral growth and eye size. Since atRA has been implicated in the regulation of postnatal ocular growth in chicks, guinea pigs, and primates [6,9,10], the purpose of this study was to examine the expression and distribution of RALDH isoforms and RALDH enzyme activity in the postnatal human eye. Results from this investigation provide the first evidence of enzymatically active RALDH in the retina, RPE, and choroid of the human eye, further emphasizing the potential importance of atRA in postnatal human eye growth and development.

## Materials and Methods

### Tissue Isolation and Preparation

RALDH activity and RALDH2 studies were undertaken several months prior to RALDH1 and RALDH3 studies, necessitating the use of two groups of human donor eyes. For RALDH activity and RALDH2 studies, posterior ocular poles were obtained from human donor eyes in the following age groups: adolescent (2 male donors, aged 16 and 17 years), young adult (two donors, aged 29 years (gender unknown), and 30 years, male), and adult (one male donor aged 46 years). For RALDH1 and RALDH3 studies, donor eyes were obtained at the following ages: 14 years (gender unknown), 49 years (male), and 50 years (female). Human donors with a history of systemic disease or chemotherapy were excluded from the study. Ocular poles were obtained from the National Disease Research Interchange’s Eye Bank (NDRI, Philadelphia, PA; ndriresource.org). Written informed consent from the donor and/or relatives was obtained by the NDRI. Eyes were received within two days of death and immediately frozen at −80°C. Human poles were handled according to the tenets of the Declaration of Helsinki. The research was approved by the University of Oklahoma Health Sciences Center’s institutional review board (IRB Number: 10783) and granted “exempt” status according to the Code of Federal Regulations, 45 CFR 46, Protection of Human Subjects.

To prepare tissues for protein extraction, poles were thawed at room temperature (RT), and the vitreous body and lens were removed. The ocular poles were cut into four equal quadrants to facilitate access to all ocular tissue layers. The neural retina was removed from each quadrant, and the RPE was separated from the choroid by gentle brushing and washing with phosphate buffered saline (PBS) (5 mM phosphate, 145 mM NaCl). PBS containing RPE was collected and pelleted by centrifugation at 10,000g. The supernatants were discarded and pellets were stored at −80°C. Following removal of RPE from the choroid layer, choroids were removed from the sclera with gentle dissection and placed into microfuge tubes on ice. The sclera was cleared of adherent extraocular muscle and placed into microfuge tubes on ice. Lenses were rinsed with PBS and placed into microfuge tubes on ice. All isolated tissues were stored at −80°C until time of use.

To prepare ocular tissue homogenates, lenses, choroids, retinas, and RPEs were separately homogenized in RALDH homogenization buffer (20 mM triethanolamine-HCl pH 7.4, 1 mM dithiothreitol, 0.1 mM EDTA) using a VirTis rotor-stator homogenizer (SP Industries, Gardiner, NY). Sclera was snap-frozen in liquid nitrogen and pulverized using a cryogenic mill (Spex 6700, Metuchen, NJ) until ground into a fine powder. The milled scleras were homogenized in homogenization buffer as described above. After homogenization, a maximum of 1.5 mL of each homogenate was placed in a thick-walled Microfuge Tube Polyallomer (Beckman Coulter, Brea, CA) and subjected to ultracentrifugation at 100,000g for 1 hr at 4°C (Optimum MAX Ultracentrifuge, Beckman Coulter, Brea, CA). Cytosol fractions (supernatant) were isolated from the pellet and stored at −80°C.

### Retinoic Acid Synthesis Assay

Retinaldehyde dehydrogenase activity was determined by measuring the production of atRA *in vitro* as previously described with minor modifications [21]. Under the conditions described, the rate of atRA synthesis is linear from 0–2 hrs using all-*trans*-retinaldehyde concentrations of 0–8 μM at 37°C. As the stoichiometry of the reaction is 1:1, the amount of all-*trans*-retinaldehyde converted to atRA during the reaction (20–200 pmoles) is at least 5 orders of magnitude less than the concentration of all-*trans*-retinaldehyde present in the reaction (25 μM).

All procedures with all-*trans*-retinaldehyde or atRA were performed in dim red light. In duplicate, 200 μL of synthesis buffer (2.5% dimethylsulfoxide, 4 mM NAD, 32 mM tetrasodium pyrophosphate pH 8.2, 0.1 mM pyrazole, 5 mM glutathione, 1 mM EDTA) was added to 200 μL cytosol from homogenized ocular tissues. The reaction was initiated by the addition of 50 μL of 250 μM all-*trans*-retinaldehyde to the reaction mixture. 200 μL of RALDH homogenization buffer was used as a blank (no cytosol present). The reactions were mixed and placed in a water bath at 37°C. After 30 min of incubation, the reaction was stopped by immersion in ice water and addition of 3/2 volumes (675 μL) of methanol. The samples were processed by HPLC, as previously described [22].

### Protein Characterization and Identification

Protein concentrations of all tissue samples were determined by a Bradford assay (BioRad, Hercules, CA). The protein concentration of each tissue sample was adjusted to a final concentration of 3.1 μg per 15 μL of dH_2_O to ensure equal protein was loaded per sample and to normalize results to total protein loaded. NuPAGE LDS Sample Buffer and NuPAGE Sample Reducing Agent were added to the 15 μL of protein so that the sample buffer and reducing agent came to a final concentration of 1X (Life Technologies, Grand Island, NY). Samples were then placed in a 70°C water bath for 10 min and electrophoresed under reducing conditions on 10% Bis-Tris Gel NuPAGE SDS-PAGE gels (Life Technologies, Grand Island, NY), according to standard protocols for the NuPAGE gel system. For visualization of total protein, gels were stained with SimplyBlue Safe Stain (Invitrogen, Grand Island, NY) according to the manufacturer’s instructions. Gel bands were quantified with ImageJ (NIH). For western blots, gels were electroblotted onto a nitrocellulose membrane (BioRad, Hercules, CA) using an electro-transfer unit (XCELL Sureback Electrophoresis Cell, Invitrogen, Grand Island, NY) according to manufacturer’s instructions. Blots were incubated in blocking buffer [0.2% I-Block (Tropix, Bedford, MA), 0.1% Tween-20 in PBS] for 1 hr at RT with gentle rocking. Blots were then incubated with rabbit anti-human RALDH2 (anti-ALDH1A2; 1:300 in blocking buffer), rabbit anti-human RALDH1 [anti-ALDH1A1 (Millipore), 1:1,000 in blocking buffer; or anti-ALDH1A1 (Sigma), 1:175 in blocking buffer], or rabbit anti-human RALDH3 (anti-ALDH1A3, 1:10,000 in blocking buffer; or anti-ALDH1A3, N-term, 1:300 in blocking buffer) overnight at 4°C (**Table 1**). Immunoblots were washed three times with PBS containing 0.05% Tween-20 followed by three washes with PBS. Immunoblots were then incubated with goat anti-rabbit IgG conjugated to alkaline phosphatase as a secondary labeling antibody (1:1,000 in PBS; BioRad, Hercules, CA). After incubation with the secondary antibody, blots were washed (as above), incubated in CDP-*Star* Ready-to-Use with Nitro-BlockII (Tropix, Bedford, MA) for 5 min, and images were captured with a Chemigenius imager (Syngene, Frederick, MD). For RALDH2 experiments, an identical blot of human samples was probed with rabbit anti-RPE65 [23] (1:1,000 in blocking buffer) followed by incubation in goat anti-rabbit IgG conjugated to alkaline phosphatase (1:1,000 in PBS). For RALDH3 experiments, 1.25 μg of purified, recombinant human RALDH3 (Sino Biologicals Inc., Beijing, China) was loaded onto the gel with the tissue samples to serve as a positive control.

**Table 1:** List of Primary Antibodies.

Antigen	Manufacturer (Catalog #)	Host
RALDH2	Sigma (HPA010022)	Rabbit
RALDH1	Sigma (HPA002123)	Rabbit
RALDH1	Merck Millipore (ABD12)	Rabbit
RALDH3, N-term	Sigma (SAB1300932)	Rabbit
RALDH3	Thermo Scientific (PA5-29188)	Rabbit

Quantification of band intensity was performed using the Manual Band Quantification feature of the Syngene GeneTools (Syngene, Frederick, MD) program. The faintest band on each image was assigned a value of 1 and all other bands were automatically assigned relative values to the faintest band. Automatic background correction was applied.

### Immunohistochemistry

Slides containing 5-micron thick paraffin sections of human posterior fundus containing the retina, RPE, choroid, and sclera were obtained from the National Disease Research Interchange (Philadelphia, PA). The donor was a 30 year old Caucasian male with a history of tobacco and marijuana use. Cause of death was not due to systemic disease.

Slides were deparaffinized in a xylene/ethanol series, washed three times in dH_2_O for 5 min, and transferred to a slide mailer (∼20 mL volume). Antigen retrieval was performed by incubating the slides in a 1% aqueous sodium borohydride solution for 2 min at room temperature, followed by washing with dH_2_O for 5 min (3X) and PBS for 5 min (3X). Following removal of the last wash, slides were incubated in sodium citrate (10 mM sodium citrate, 0.05% Tween-20, in dH_2_O at pH 6.0) for 20 min in a 95°C water bath. The water bath was turned off, and the slide mailer remained in the bath for an additional 20 min. The mailer was removed from the water bath, allowed to cool to RT, and slides were washed with PBS for 5 min (3X). Slides were incubated in blocking buffer (2% BSA, 0.2% Triton X-100, in PBS pH 7.4) for 1 hr at RT with rocking, and then incubated in blocking buffer containing rabbit anti-RALDH2 (1:300) or rabbit anti-RALDH1 (1:100) for 5 days at 4°C (**Table 1**). Slides were washed with PBS for 10 min (6X), incubated with goat anti-rabbit IgG conjugated to AlexaFluor 568 (1:1000 in PBS; Life Technologies Grand Island, NY) for 2 hrs at RT, and washed with PBS for 10 min (6X). Slides were then incubated with the nuclear stain, 4',6-diamidino-2-phenylindole (DAPI; 5 μg/mL in dH_2_O) for 5 min and coverslipped using ProLong Gold Antifade reagent containing DAPI (Invitrogen, Grand Island, NY). For double labeling experiments, after incubation with anti-RALDH2, goat anti-rabbit IgG- AlexaFluor 568 conjugate, and subsequent washes, slides were incubated with mouse anti-α-smooth muscle actin (1:50 dilution in blocking buffer; Sigma, St. Louis, MO) for an additional 5 days at 4°C. Slides were then washed (described above) and incubated with rabbit anti-mouse IgG conjugated to AlexaFlour 488 (1:1000 in PBS; Life Technologies, Grand Island, NY) for 2 hrs. Slides were washed and mounted as described above and viewed using a Fluoview FV1000 confocal laser scanning microscope (Olympus America Inc., Center Valley, PA).

The following were used for negative controls: 1) primary antibody was omitted from the first incubation; 2) tissue sections were incubated in 10 μg/mL non-immune rabbit IgG (Sigma, St. Louis, MO) instead of the primary antibody. Additional preabsorption controls were performed in which the anti-RALDH2 antibody was incubated for 30 min at RT with 33.7 nM of purified recombinant chicken RALDH2 (Harper, *et*. *al*., manuscript in preparation) before immunolabeling paraffin sections of human eyes.

### Statistics

Analyses between groups were made using a one-way ANOVA followed by the Tukey-Kramer test for multiple comparisons (GraphPad Prism ver.5, La Jolla, CA).

## Results

### Retinaldehyde Dehydrogenase (RALDH) Activity in Human Ocular Tissue

**Fig 1:**
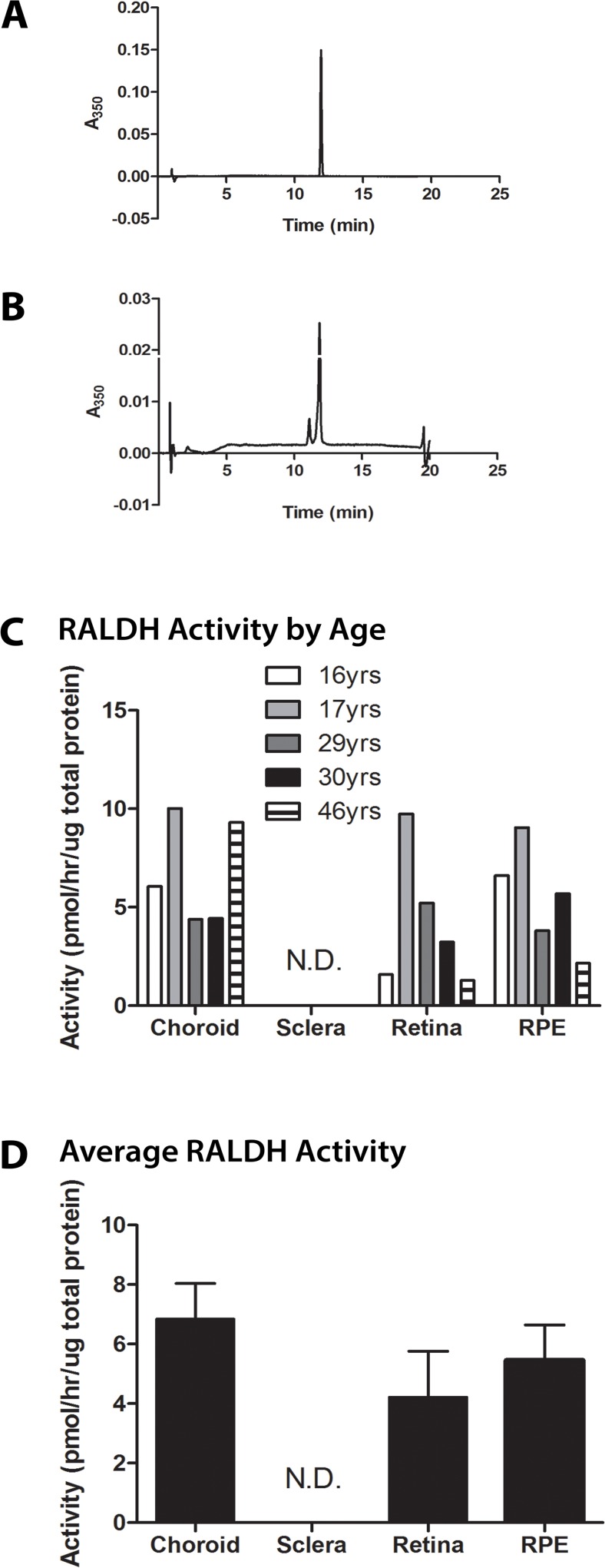
RALDH catalytic activity in postnatal human ocular tissues using a HPLC/spectrophotometric assay for NAD-dependent atRA synthesis. **(A)** Chromatogram of pure atRA (425 pmol), demonstrating that a pure solution of atRA elutes at 12 minutes. **(B)** Representative chromatogram of a choroid cytosol fraction from a 46 year old donor. The major peak, eluting at approximately 12 minutes, contained 171 pmol of atRA, corresponding to a rate of 155 pmol/minute/mg protein. **(C)** RALDH activity in ocular tissues of donors aged 16–46 years. **(D)** Average RALDH activity (± s.e.m.) in the ocular tissues of the ages presented in (C) (n = 5). Graphs in (C-D) were normalized to the total protein concentration in the cytosol fractions of each tissue sample. Each activity measurement represents the average of two duplicate samples. RALDH catalytic activity in the sclera was below the level of detection in all samples.

Since results from experimental animal models have suggested that postnatal ocular tissues can synthesize all-*trans*-retinoic acid (atRA) from all-*trans*-retinaldehyde via RALDH enzymes [7], RALDH enzymatic activity was examined in postnatal human ocular tissues. The rate of atRA synthesis from all-*trans*-retinaldehyde was measured in cytosol fractions of ocular tissues from each donor (**Fig. 1**). Following 30 minutes of incubation of tissue cytosol fractions with all-*trans*-retinaldehyde in the presence of NAD, atRA could be detected by HPLC as a sharp peak eluting at approximately 12 minutes (**Fig. 1A, 1B**). This peak was not detected in samples incubated in the absence of tissue homogenate or in tissue homogenates incubated in the absence of NAD or all-*trans*-retinaldehyde, indicating all detectible atRA was synthesized by ocular tissues *in vitro*. All results were normalized to the total protein present in each cytosol fraction. RALDH activity could be detected in the retina, RPE, and choroid in all ages examined with activity ranging from 1.3–9.7 pmol/hr/μg total protein in the retina, 2.2–9.0 pmol/hr/μg total protein in the RPE, and 4.4–10.0 pmol/hr/μg total protein in the choroid (**Fig. 1C**). RALDH activity in the sclera was below the level of detection. It is interesting to note the relatively high RALDH activity in the retina of the 17 year old donor compared to the 16 year old donor. We speculate that postnatal differences in RALDH activity may underlie differences in ocular growth and refraction; however, since a detailed ocular history was not provided with the donor eyes, the interpretation of these results is limited. When averaged for all donors (**Fig. 1D**), RALDH activity was somewhat higher in the choroid (6.84 ± 1.20 pmol/hr/μg total protein), followed by the RPE (5.46 ± 1.18 pmol/hr/μg total protein), and retina (4.21 ± 1.55 pmol/hr/μg total protein), although differences were not statistically significant (p = .5385).

### RALDH2 Expression in Human Ocular Tissue

**Fig 2:**
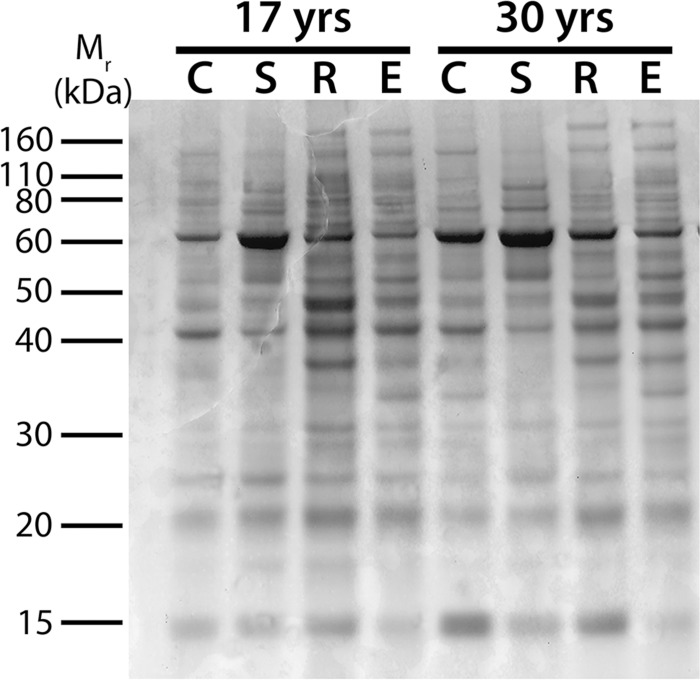
SDS-PAGE minigel of cytosol fractions from human ocular tissues. 3 μg total protein from choroid (C), sclera (S), retina (R), and RPE (E) cytosol fractions (100,000g supernatants) from human donors aged 17 and 30 years were separated on a 1.0 mm 10% Bis/Tris gel.

In order to determine which RALDH isoforms could be contributing to the RALDH activity observed above, the presence of each RALDH isoform in postnatal human ocular tissues was examined. To ensure equal loading of protein (3 μg/lane), representative samples of cytosol from choroid, sclera, retina, and RPE were selected from each age group, separated by SDS-PAGE, and stained with Coomassie Blue (**Fig. 2**; see **S1 Fig.** for full gel). As expected, Coomassie Blue staining indicated distinct differences in protein profiles between cytosol fractions of each tissue type, but quantification of band intensities confirmed that similar amounts of total protein were loaded for each sample. In addition, minimal degradation was observed between donors based on the presence of distinct bands migrating between 10 and 200 kDa.

**Fig 3:**
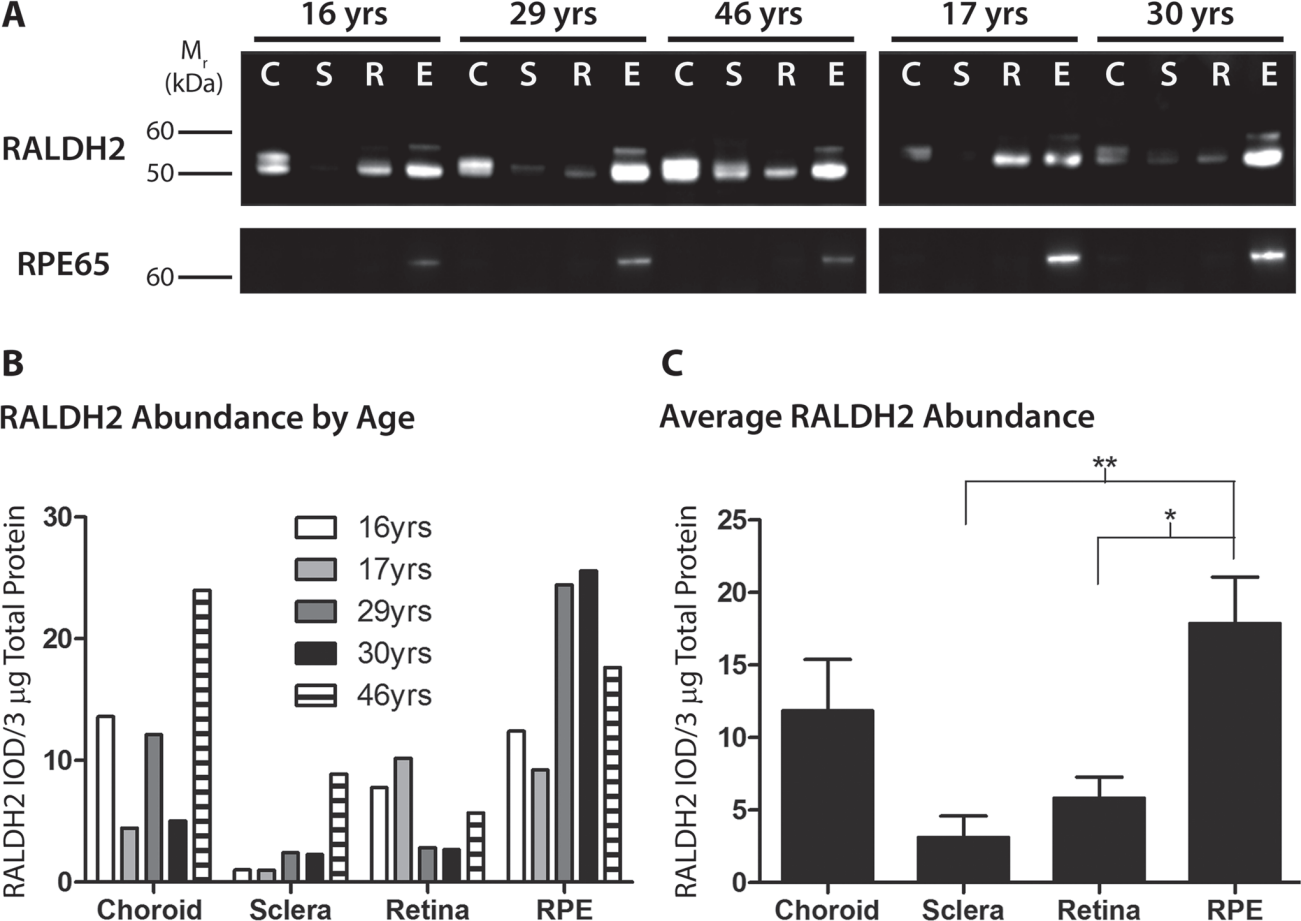
Western blot analysis and quantification of RALDH2 in postnatal human ocular tissues. **(A)** Cytosol fractions from ocular tissues of donors aged 16–46 years were immunblotted with anti-RALDH2 or anti-RPE65. *Top Panel*: RALDH2 immunopositive bands (∼ 55–59 kDa) were present in the choroid (C), sclera (S), retina (R), and RPE (E) of postnatal human eyes. *Bottom Panel*: anti-RPE65 was used to identify the presence of RPE contamination in the retina, choroid, and sclera samples. 3 μg total protein/lane was loaded for each blot. **(B)** Relative abundance of RALDH2 was measured as the integrated optical density (IOD) per 3 μg total protein of RALDH2-immunopositive bands. **(C)** Average RALDH2 abundance (± s.e.m.) in ocular tissues of all donors presented in (B) (n = 5). *p < 0.05, **p < 0.01 (one-way ANOVA followed by Tukey-Kramer test for multiple comparisons).

As RALDH2 has recently been identified as synthesizing atRA in the postnatal chick choroid in response to visually guided eye growth [7], the presence of this isoform was investigated first. Western blot analysis of postnatal human ocular tissues with anti-RALDH2 antibodies showed that RALDH2 could be detected in varying abundance in all postnatal ocular tissues and all age groups examined (**Fig. 3**). All results were normalized to total protein present in each cytosol fraction. In choroid samples, RALDH2 protein migrated as a closely spaced doublet with bands at ∼57 and 55 kDa. In retina and RPE samples, RALDH2 was present as a predominant band at 55 kDa together with a minor immunopositive band at ∼59 kDa (**Fig. 3A**, top panel). RALDH2 was also detected in the human sclera as a relatively faint band migrating at ∼55 kDa. It is suspected that the variations in size of the RALDH2 immunopositive bands observed within and among the tissues is due to various splice variants of the RALDH2 protein. Human RALDH2 has four known splice variants with molecular weights ranging from 46.1 to 56.7 kDa. (See **S2 Fig.** for full blot.)

To evaluate potential RPE contamination in the retina, choroid, and sclera, identical blots were probed using an antibody for RPE65, an isomerahydrlase specific to the RPE that converts all-*trans*-retinyl esters to 11-*cis*-retinol (**Fig. 3A**, bottom panel). Following immunoblotting with anti-RPE65, RPE65 could be readily detected in the RPE of every donor, with a singular band found at ∼65 kDa. (See **S3 Fig.** for full blot.)

Relative RALDH2 abundance in human ocular tissue samples was determined by digital image analysis of band intensities on western blots in order to compare the relative tissue distribution of RALDH2 in the postnatal human eye (**Fig. 3B, 3C**). Comparisons of relative RALDH2 abundance in human ocular tissue samples were reported as 1) “RALDH2 abundance by age” (**Fig. 3B**), where the integrated optical density (IOD) per 3 μg total protein for RALDH2 immunopositive bands was reported for each individual sample; and 2) “average RALDH2 abundance” (**Fig. 3C**), where values from Fig. 3B for each tissue were averaged across all donors (n = 5 donors for each tissue). RALDH2 appeared to be highest in relative abundance in the RPE, followed by the choroid, retina, and had the lowest relative abundance in the sclera (**Fig. 3B**). When the absolute levels of RALDH2 (values from Fig. 3B) were averaged across all age groups (**Fig. 3C**), RALDH2 showed the same overall pattern of distribution across the tissues types; RALDH2 (RALDH2 IOD/3 μg total protein) was highest in the RPE (17.86 ± 3.22), followed by the choroid (11.83 ± 3.55), retina (5.83 ± 1.44), and lowest in the sclera (3.11 ± 1.48). Significant differences in RALDH2 density were observed between the RPE and sclera (*p* < 0.01, ANOVA followed by Tukey-Kramer for multiple comparisons) and between the RPE and retina (*p* < 0.05, ANOVA followed by Tukey-Kramer for multiple comparisons).

### Cellular Localization of RALDH2

**Fig 4:**
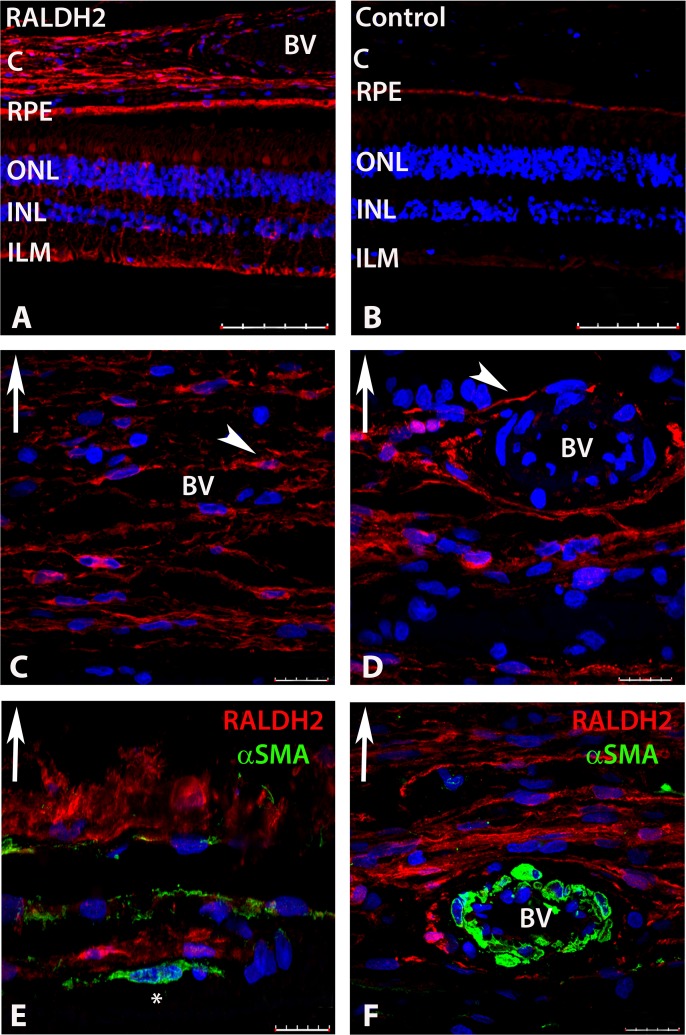
Confocal images of RALDH2 *(red)* and αSMA *(green)* expressing cells after immunolabeling with anti-RALDH2 and anti-αSMA. **(A)** Low power magnification of ocular tissues demonstrating RALDH2 labeling in the retina, RPE, and choroid. **(B)** Negative control section (primary antibody pre-absorbed with recombinant RALDH2) demonstrating absence of labeling in the retina and choroid and slight auto-fluorescence in the RPE. **(C, D)** Choroid demonstrating RALDH2 labeling in extravascular cells closely associated with blood vessels and in slender cells throughout the stroma (arrowhead). **(E, F)** Double labeling of the choroid with anti-RALDH2 and anti-αSMA demonstrates RALDH2 positive cells are distinct from vascular and extravascular smooth muscle cells. Asterisk (*) indicates an αSMA-positive extravascular smooth muscle cell in (E). Upward arrow in (C-F) indicates orientation for the scleral side of the choroid tissue. Nuclei were counterstained with DAPI (*blue)*. BV, blood vessel; C, choroid; RPE, retinal pigment epithelium; ONL, outer nuclear layer; INL, inner nuclear layer; ILM, inner limiting membrane. Scale bars = 100 μm in A, B; 20 μm in C-F.

Immunohistochemistry was performed on sections of human posterior ocular tissues (retina, RPE, choroid) in order to determine the cellular localization of RALDH2 and identify the cells responsible for atRA synthesis in these tissues (**Fig. 4**). When RALDH2 was probed in the postnatal human eye with an anti-human RALDH2 antibody followed by a secondary antibody conjugated to AlexFlour 568 (red), RALDH2 could be detected in the retina, choroid, and RPE (**Fig. 4A**). Based on morphology and position in the retina, RALDH2 positive cells in the retina were speculated to be Müller cells and cone outer segments. No labeling of the retina or choroid was found in negative controls in which primary antibody was pre-incubated with recombinant RALDH2 prior to immunolabeling (**Fig. 4B**), when non-immune rabbit IgG was used in place of the primary antibody, or when sections were not exposed to primary antibody. However, some non-specific labelling was detected in the RPE and is most likely due to autofluorescence of RPE-associated lipofuscin [24], as fluorescence was still observed in the RPE in tissue sections of human eyes following deparaffinization, blocking, washing, and DAPI treatment (no primary or secondary antibodies were used).

In the choroid, RALDH2 was found in attenuated cells in the choroidal stroma (**Fig. 4A**), some of which were closely associated with the exterior of small blood vessels (**Fig. 4C, 4D**). In order to determine if the RALDH2-positive cells were vascular and/or extravascular smooth muscle, slides were double labeled with anti-RALDH2 together with anti-α-smooth muscle actin (**Fig. 4E, 4F**). RALDH2 did not co-localize with either non-vascular (**Fig. 4E**) or vascular (**Fig. 4F**) smooth muscle cells.

### RALDH1 Expression in Human Ocular Tissue

**Fig 5:**
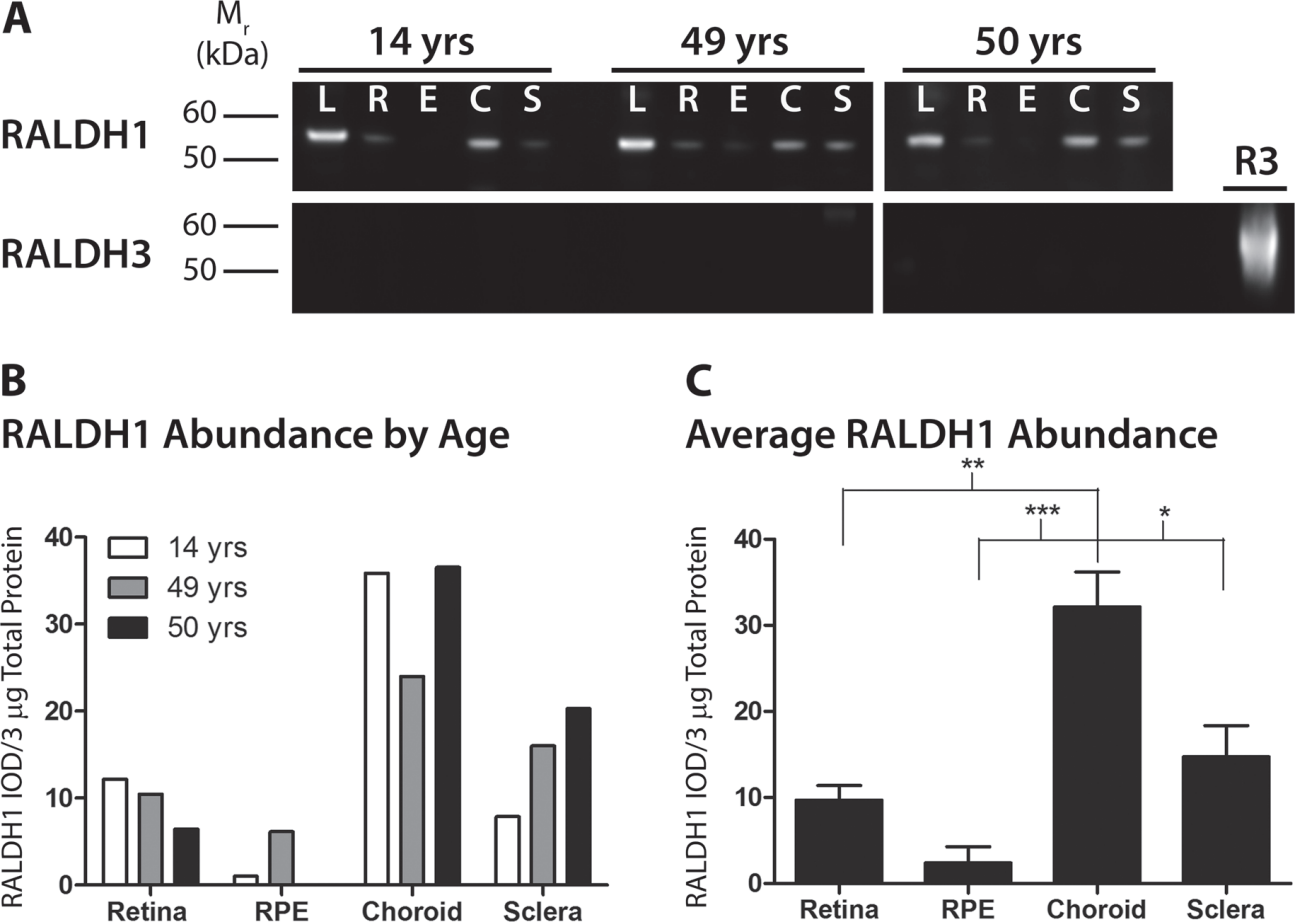
Western blot analysis and quantification of RALDH1 and RALDH3 in postnatal human ocular tissues. **(A)** Cytosol fractions from ocular tissues of donors aged 14, 49, and 50 years were immunblotted with anti-RALDH1 or anti-RALDH3. *Top panel*: RALDH1 (∼ 55–57 kDa) was abundantly expressed in the lens and choroid, moderately expressed in the sclera, and faintly detected in the retina and RPE. *Bottom Panel*: RALDH3 was not detected in postnatal ocular tissues at any of the ages examined. 3 μg total protein/lane was loaded on the blot. 1.25 μg recombinant human RALDH3 (R3) was loaded as a positive control. **(B)** Abundance of RALDH1 was measured as the integrated optical density (IOD) per 3 μg total protein of RALDH1-immunopositive bands. **(C)** Average RALDH1 abundance (± s.e.m.) in ocular tissues of all donors presented in (B) (n = 3). *p < 0.05, **p < 0.01, ***p < 0.001 (one-way ANOVA followed by Tukey-Kramer test for multiple comparisons).

Western blotting was employed to determine if the RALDH1 isoform was also present in postnatal human ocular tissues (**Fig. 5A**). Following labeling with anti-RALDH1 antibodies, RALDH1 was detected faintly in the retina and RPE, moderately expressed in the sclera, and strongly expressed in the choroid at all age groups examined. RALDH1 protein migrated as a single band at ∼55 kDa in the retina and RPE and at ∼57 kDa in the choroid and sclera (**Fig. 5A,** top panel). As RALDH1 has previously been shown to be abundantly expressed in the lens [25,26], cytosol fractions of human lens was used as a positive control for RALDH1 labeling. In the lens, RALDH1 was detected strongly at ∼55 kDa. (See **S4 Fig.** for full blot.) In addition, RALDH3 protein expression was evaluated in postnatal human ocular tissues by western blot using antibodies specific for the RALDH3 isoform. No immunopositive bands were detected in postnatal human ocular tissues by western blot (**Fig. 5A,** bottom panel). Recombinant, purified human RALDH3 was employed as a positive control (**Fig. 5A,** lane “R3”). (See **S5 Fig.** for full blot.)

Similar to RALDH2 analyses, relative RALDH1 abundance in human ocular tissue samples was determined by digital image analysis of band intensities and was reported as 1) “RALDH1 abundance by age” (**Fig. 5B**); and 2) “average RALDH1 abundance” (**Fig. 5C**). RALDH1 appeared to be highest in relative abundance in the choroid, in moderate abundance in the sclera, and was present at relatively low levels in the retina and RPE (**Fig. 5B**). When the absolute levels of RALDH1 (values from Fig. 5B) were averaged across all age groups (n = 3 donors for each tissue) (**Fig. 5C**), average RALDH1 (RALDH1 IOD/3 μg total protein) abundance was highest in the choroid (32.13 ± 4.07) with moderate levels in the retina (9.68 ± 1.70) and sclera (14.73 ± 3.63) and lowest levels in the RPE (2.39 ± 1.91). Significant differences in RALDH1 density were observed between the choroid and retina (*p* < 0.01, ANOVA followed by Tukey-Kramer for multiple comparisons), the choroid and RPE (*p* < 0.001, ANOVA followed by Tukey-Kramer for multiple comparisons), as well as the choroid and sclera (*p* < 0.05, ANOVA followed by Tukey-Kramer for multiple comparisons).

### Cellular Localization of RALDH1

**Fig 6:**
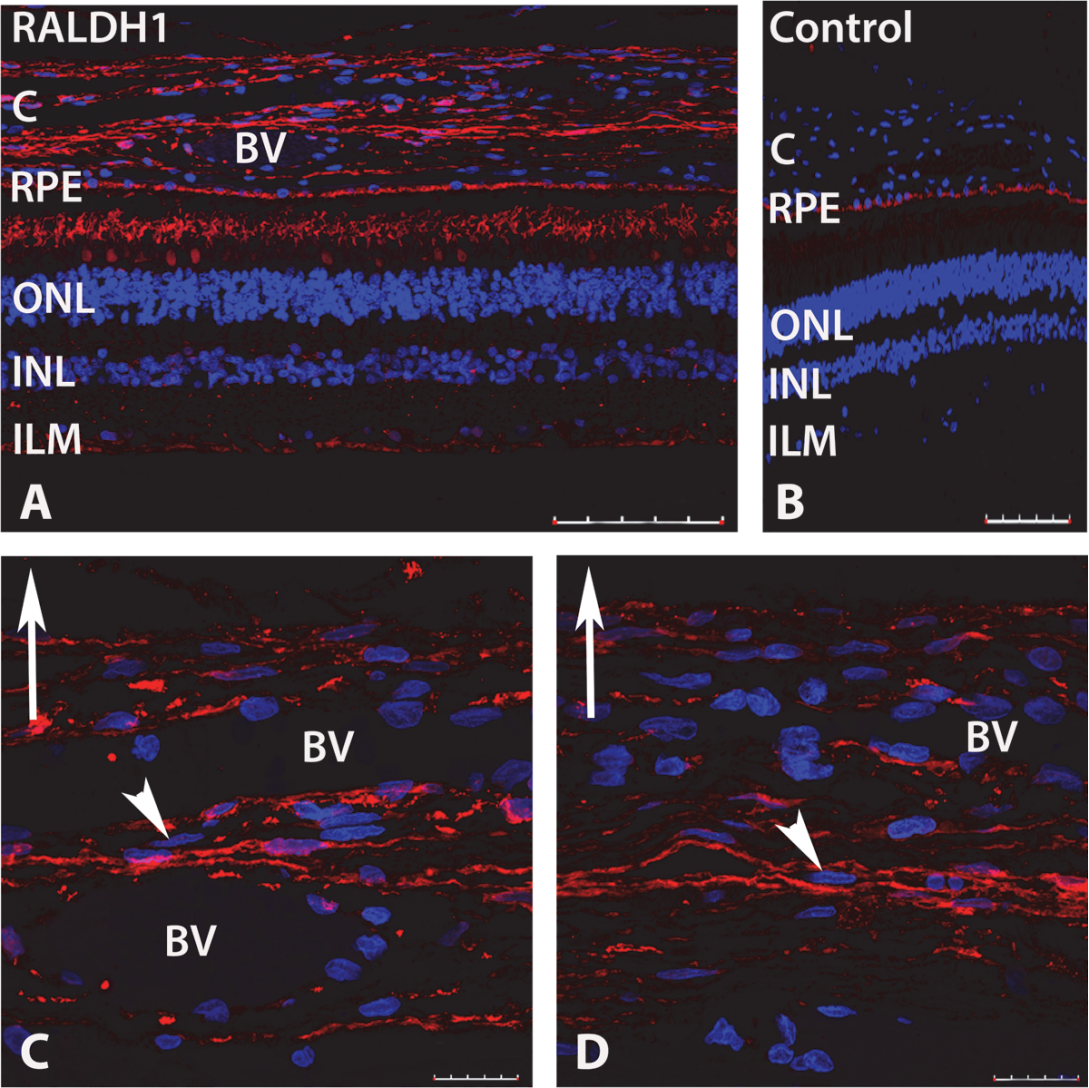
Confocal images of RALDH1 expressing cells in postnatal human ocular tissue after immunolabeling with an anti-RALDH1 antibody. **(A)** Low power magnification of ocular tissues demonstrating RALDH1 labeling *(red)* in the retina and choroid. **(B)** Negative control slide (non-immune IgG used in place of primary antibody) demonstrating no labeling in the retina and choroid and auto-fluorescence in the RPE. **(C, D)** Choroid sections demonstrating RALDH1 labeling in extravascular cells throughout the choroidal stroma (arrowhead). Upward arrow in (C-D) indicates orientation for the scleral side of the choroid. Nuclei were counterstained with DAPI (*blue)*. BV, blood vessel; C, choroid; RPE, retinal pigment epithelium; ONL, outer nuclear layer; INL, inner nuclear layer; ILM, inner limiting membrane. Scale bars = 100 μm in A, B; 20 μm in C, D.

In order to determine the cellular localization of RALDH1, immunohistochemistry was performed on sections of human posterior ocular tissues (retina, RPE, choroid) (**Fig. 6**). When sections of human fundus were probed with anti-human RALDH1 followed by anti-rabbit IgG conjugated to AlexFlour 568 (red), RALDH1 could be detected in the retina and choroid (**Fig. 6A**). Based on morphology and placement in the retina, RALDH1 immunopositive labeling was observed in rod outer segments and a subpopulation of cone outer segments. Additionally, punctate labelling could be detected throughout the inner plexiform layer and inner limiting membrane, possibly associated with Müller cells. No labeling of the retina or choroid was found in negative controls in which non-immune IgG was used as the primary antibody (**Fig. 6B**). Non-specific labelling was detected in the RPE and is most likely due to autofluorescence of RPE-associated lipofuscin (discussed above) [24].

In the choroid, strong RALDH1 labeling was found in extravascular cells throughout the choroidal stroma (**Fig. 6C, 6D**). Unlike RALDH2 labeling, RALDH1 labeling did not appear to associate with the exterior of blood vessels (**Fig. 6C, 6D**).

## Discussion

atRA is known to be physiologically synthesized by three cytosolic retinal dehydrogenases (RALDH1, RALDH2 and RALDH3), which belong to the aldehyde dehydrogenase superfamily [27]. Because each of the RALDH isoforms mediates the irreversible conversion of all-*trans*-retinaldehyde to atRA, the presence of RALDH indicates where atRA is being produced. RALDH2 has previously been shown to be the most abundant RALDH isoform expressed in the postnatal chick choroid and was localized to a unique subpopulation of extravascular choroidal stromal cells [7]. Moreover, choroidal RALDH2 expression has been shown to be modulated by changes in the visual environment, where it is presumed to regulate atRA synthesis and downstream changes in scleral extracellular matrix remodeling. Therefore, RALDH2, through its synthesis of atRA, is an attractive therapeutic target for control of scleral remodeling in human conditions such as myopia. To determine whether results originally obtained in lower vertebrate species could potentially be translated to humans, RALDH enzyme activity and relative protein abundance of the major RALDH isoforms were examined in ocular fundal tissues from human donor eyes aged 14–50 years.

### RALDH Activity

RALDH activity was detected in cytosol fractions of choroid, retina, and RPE of all donors. The choroid, retina, and RPE synthesized atRA from all-*trans*-retinaldehyde in the presence of NAD at rates ranging from 0.5–10.0 pmol/hr/μg total protein. Average RALDH catalytic activities were similar for choroid, retina, and RPE; 6.84 ± 1.20, 4.21 ± 1.55, and 5.46 ± 1.18 pmol/hr/μg total protein, respectively. Interestingly, the rates of RALDH catalytic activity measured from postnatal human ocular tissues are higher than those reported for the embryonic chick eye at 6 days of development, of which retina cytosol was reported to be the most abundant enzyme source (0.76 ± 0.053 pmol/hr/μg total protein) [28]. Although differences in assay conditions between the human and chick studies may account for some variability in measurements of RALDH activity, our results demonstrate, for the first time, the presence of catalytically active RALDH in adult human retina, RPE, and choroid and suggest that these tissues have the ability to synthesize atRA *in vivo*. *In vivo*, however, RALDH activity is highly dependent on a number of factors including substrate and cofactor availability, the presence of cytosolic inhibitors, the rate of substrate turnover, and the presence of retinoid specific binding proteins, which may differ considerably from the assay conditions used in the present study. Therefore, extrapolation of RALDH activities determined *in vitro* from isolated human ocular tissues obtained postmortem to true RALDH activities in the postnatal human eye should only be done with caution.

### RALDH Isoform Expression in Human Fundal Tissues

Based on the potential role of choroidal RALDH2 in modulating postnatal ocular growth, our initial studies examined relative RALDH2 abundance and distribution in the retina, RPE, choroid, and sclera of 5 human donor eyes, of ages 16–46 years. Variation in RALDH2 protein abundance was noted in ocular tissues between donors, either due to age-related or phenotypic differences between individual donors. However, when RALDH2 concentrations in each ocular tissue were averaged for all donors, significant differences in relative abundance could be detected. RALDH2 was greatest in relative abundance in the RPE of the postnatal human eye, with lesser amounts in the choroid, and lowest relative abundance in the sclera and the neural retina. These results are in contrast to those obtained from chick eyes, in which little to no RALDH2 was present in the postnatal chick RPE [7]. However, McCaffery *et al*. showed that in response to light, the postnatal murine RPE demonstrates RALDH activity and atRA synthesis above that of other ocular tissues [29]. This light mediated atRA synthesis was determined to be the result of diffusion of the all-*trans*-retinaldehyde substrate from the photoreceptors following light-induced isomerization of 11-*cis*-retinaldehyde into the RPE, followed by oxidation of all-*trans*-retinaldehyde to atRA by RALDH. Although the specific RALDH isoform responsible for light-mediated atRA synthesis in the mouse RPE was not identified, our results suggest that RALDH2 is present in the human RPE where it may catalyze light-mediated atRA synthesis from all-*trans*-retinaldehyde most likely derived from the photoreceptors as a component of the light regulated retinoid fluxes between the retina and RPE [30].

In the choroid, RALDH2 was localized to a population of extravascular stromal cells in close association with vascular smooth muscle cells, as well as in attenuated cells throughout the choroidal stroma. In contrast to previous immunolocalization studies in chick [7], RALDH2 was not expressed in extravascular smooth muscle cells in the human choroid. Research is ongoing in our laboratory to positively identify the RALDH2 immunopositive cells in the chick and human choroid.

RALDH1 protein expression was also observed in the human choroid, comparable in abundance to that observed in the lens, with moderate expression in the sclera, and substantially lower expression in the retina and RPE. In the choroid, RALDH1 immunopositive cells were identified as attenuated cells in the stroma, similar in appearance to that of RALDH2 positive cells. RALDH3 was undetectable in postnatal human ocular tissues. Interestingly, in contrast to results obtained in humans, RALDH1 mRNA expression was undetectable in the chick choroid [7]; only RALDH2 and RALDH3 mRNA could be detected by RT-qPCR. Taken together, the results of the present study indicate that RALDH activity in the human choroid is mediated by both RALDH1 and RALDH2 isoforms. However, the relative contribution of each RALDH isoform to the total catalytic activity observed in the human choroid could not be determined. Of note, no RALDH activity was detected in the sclera despite detectible (albeit low) levels of scleral RALDH1 and RALDH2 by western blotting. Since RALDH1 and RALDH2 contribute to the overall RALDH activity in the sclera, the sclera could be a potential source of atRA. However, the detection of atRA synthesis by the sclera using our *in vitro* assay conditions may be beyond the limits of detection with this assay. In addition, it is possible that cytosolic inhibitors are present in the scleral lysates that inhibit RALDH activity in our *in vitro* assay.

### Potential Roles of RALDH in the Postnatal Eye

During embryogenesis, RALDH2, together with cytochrome CYP26A1, establish local atRA gradients that facilitate a number of developmental processes including neurogenesis, cardiogenesis, body axis extension, and limb, foregut, and eye development [31–35]. The role of the RALDH isozymes and atRA in postnatal growth and development is less well-understood. However, an increasing number of studies suggest that many of the same functions that atRA directs in the embryo are involved in the regulation of growth, regeneration, and plasticity of adult organ systems [36–42]. The present study identifies RALDH catalytic activity and RALDH1 and RALDH2 protein in the retina, RPE, and choroid of postnatal human eyes. In the human RPE and retina, RALDH2 may function to oxidize excess all-*trans*-retinaldehyde, produced by photoreceptors during the visual cycle, as previously described for the mouse eye [29]. This light-induced atRA would then be available to regulate a variety of fundamental cellular processes in the retina and RPE at the transcriptional level. The presence of RALDH1 and RALDH2 in the choroid suggests that the choroid is an additional site of atRA synthesis required for visual and/or non-visually related functions. Choroidally-derived atRA may participate in the maintenance of the choroidal neurons, stromal cells or vasculature, or may regulate transcriptional activity in the neighboring RPE or sclera. In support of a role for choroidally-derived atRA in scleral growth and maintenance, we and others have previously shown that in chicks and primates, atRA is a potent regulator of scleral proteoglycan synthesis and that the choroid generates local atRA concentrations sufficient to inhibit scleral proteoglycan synthesis [6,7,9]. We therefore hypothesize that through its activity on the sclera, choroidally-generated atRA may represent the final step in the retinal-to-scleral chemical cascade involved during periods of visually guided ocular growth. Results of the present study demonstrate RALDH1 and RALDH2 protein expression and RALDH enzymatic activity in the postnatal human choroid, retina, and RPE. We speculate that through the generation of atRA, expression of RALDH1 and RALDH2 may play a role in postnatal ocular growth in humans, similar to that of atRA in visually guided eye growth of the chick and other mammalian species.

## Supporting Information

S1 Fig Full SDS-PAGE gel for Coomassie Blue staining (Fig. 2). All data gathered from the 47 year old donor was excluded from analyses due to significant RPE65 contamination in the choroid of this donor (see **S3 Fig.**). C, choroid; S, sclera; R, retina; E, RPE.
(TIF)

S2 Fig Full western blot for RALDH2 immunolabeling (Fig. 3A, top panel). Molecular marker is shown in bright field, while the full western blot is shown as a chemi-luminescent image. C, choroid; S, sclera; R, retina; E, RPE.
(TIF)

S3 Fig Full western blot for RPE65 immunolabeling (Fig. 3A, bottom panel). Molecular marker is shown in bright field, while the full western blot is shown as a chemi-luminescent image. All data gathered from the 47 year old donor was excluded from analyses due to significant RPE65 contamination in the choroid of this donor. C, choroid; S, sclera; R, retina; E, RPE.
(TIF)

S4 Fig Full western blot for RALDH1 immunolabeling (Fig. 5A, top panel). Molecular marker is shown in bright field, while the full western blot is shown as a chemi-luminescent image. L, lens; R, retina; E, RPE; C, choroid; S, sclera.
(TIF)

S5 Fig Full western blot for RALDH3 immunolabeling (Fig. 5A, bottom panel). Molecular marker is shown in bright field, while the full western blot is shown as a chemi-luminescent image. L, lens; R, retina; E, RPE; C, choroid; S, sclera. R3, recombinant human RALDH3. Higher molecular weight bands in the “R3” lane are RALDH3 oligomers.
(TIF)
