# Supplementary figures and images for: Identification of Active Retinaldehyde Dehydrogenase Isoforms in the Postnatal Human Eye
Source: PLoS One. 2015 Mar 20;10(3):e0122008. doi: 10.1371/journal.pone.0122008 (PMC4368790; doi:10.1371/journal.pone.0122008)

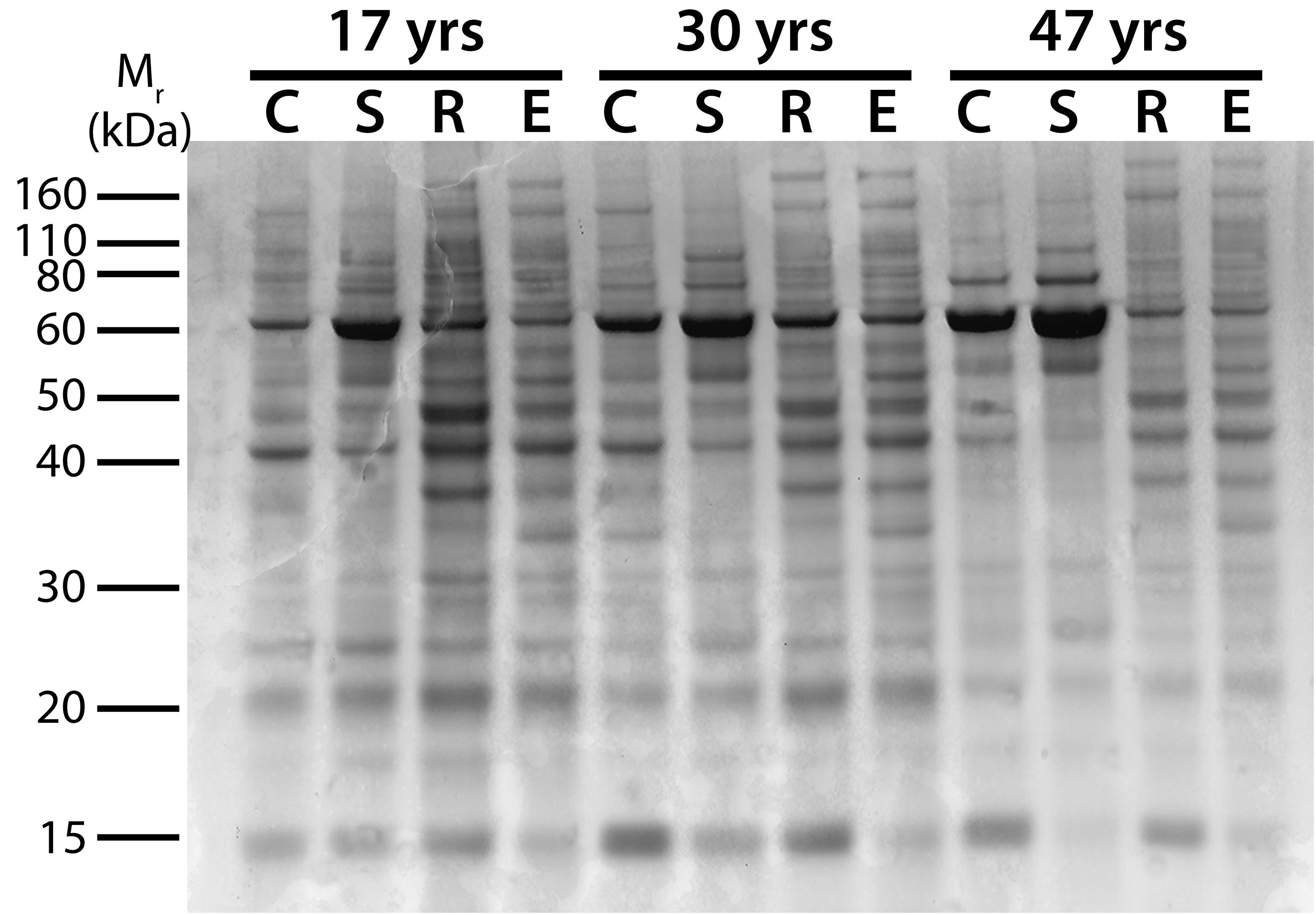

Supplement: S1 Fig — All data gathered from the 47 year old donor was excluded from analyses due to significant RPE65 contamination in the choroid of this donor (see S3 Fig.). C, choroid; S, sclera; R, retina; E, RPE. (TIF) [file pone.0122008.s001.tif]

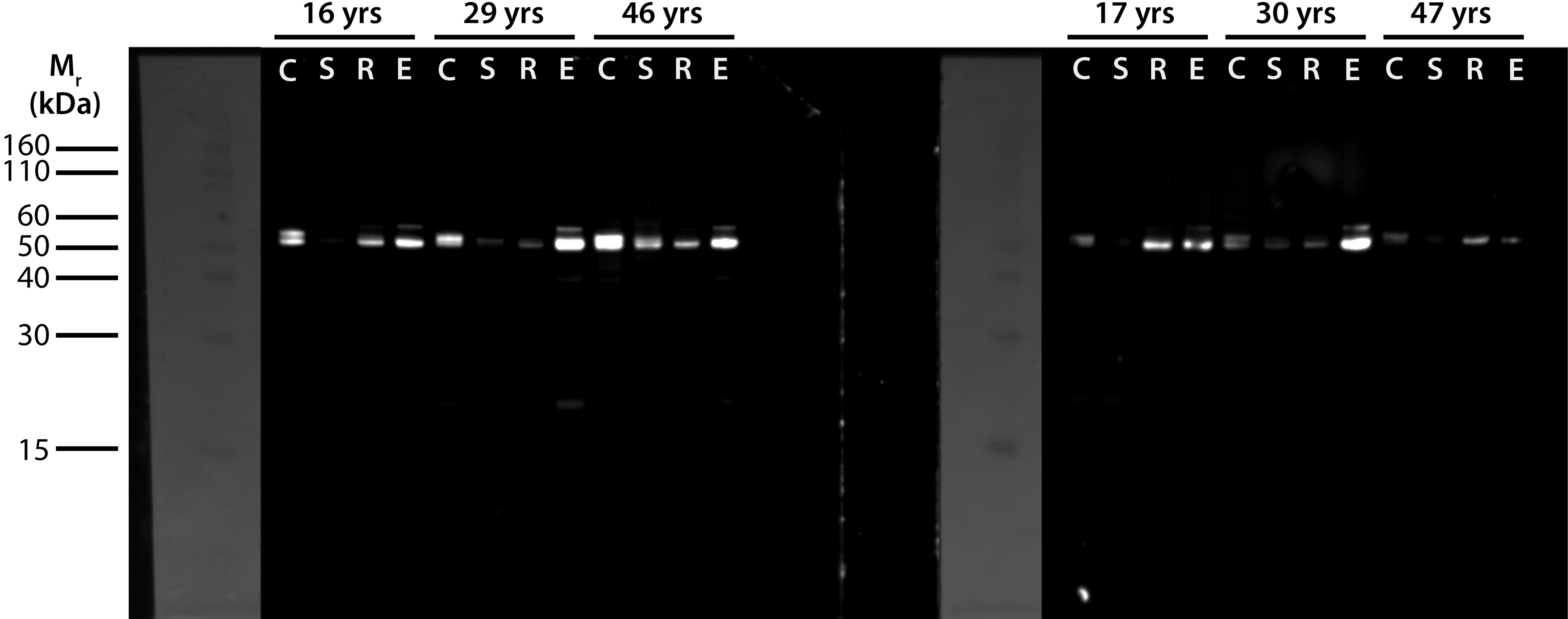

Supplement: S2 Fig — Molecular marker is shown in bright field, while the full western blot is shown as a chemi-luminescent image. C, choroid; S, sclera; R, retina; E, RPE. (TIF) [file pone.0122008.s002.tif]

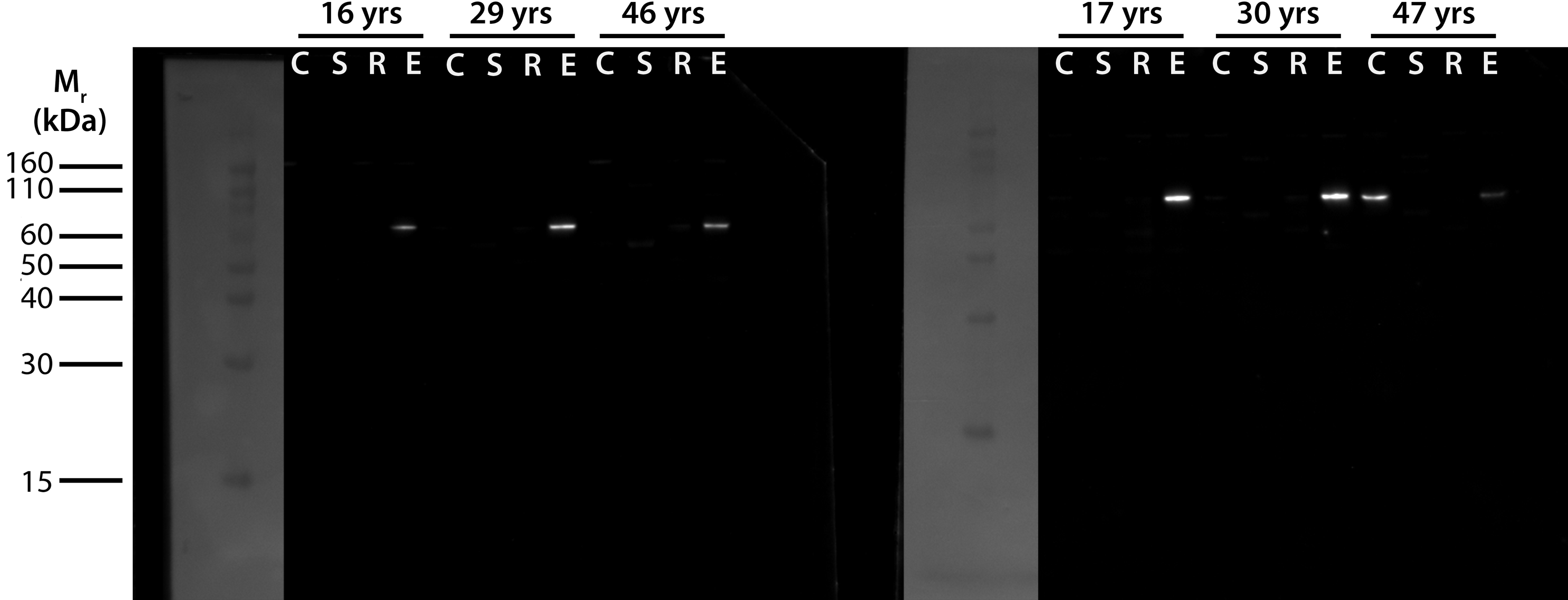

Supplement: S3 Fig — Molecular marker is shown in bright field, while the full western blot is shown as a chemi-luminescent image. All data gathered from the 47 year old donor was excluded from analyses due to significant RPE65 contamination in the choroid of this donor. C, choroid; S, sclera; R, retina; E, RPE. (TIF) [file pone.0122008.s003.tif]

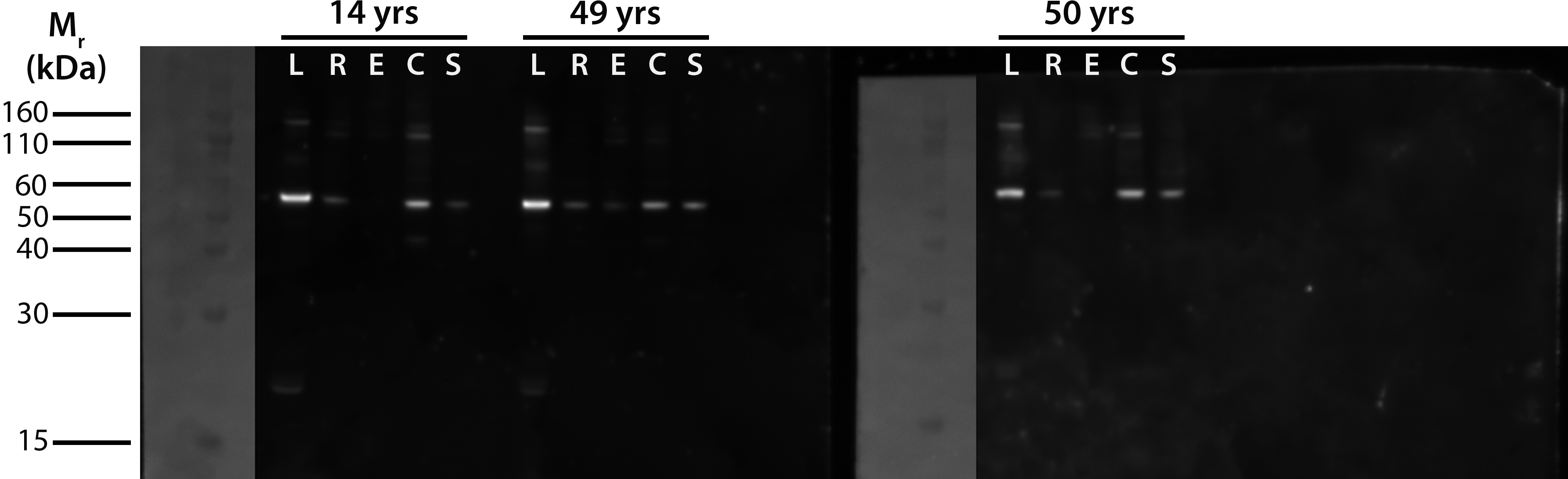

Supplement: S4 Fig — Molecular marker is shown in bright field, while the full western blot is shown as a chemi-luminescent image. L, lens; R, retina; E, RPE; C, choroid; S, sclera. (TIF) [file pone.0122008.s004.tif]

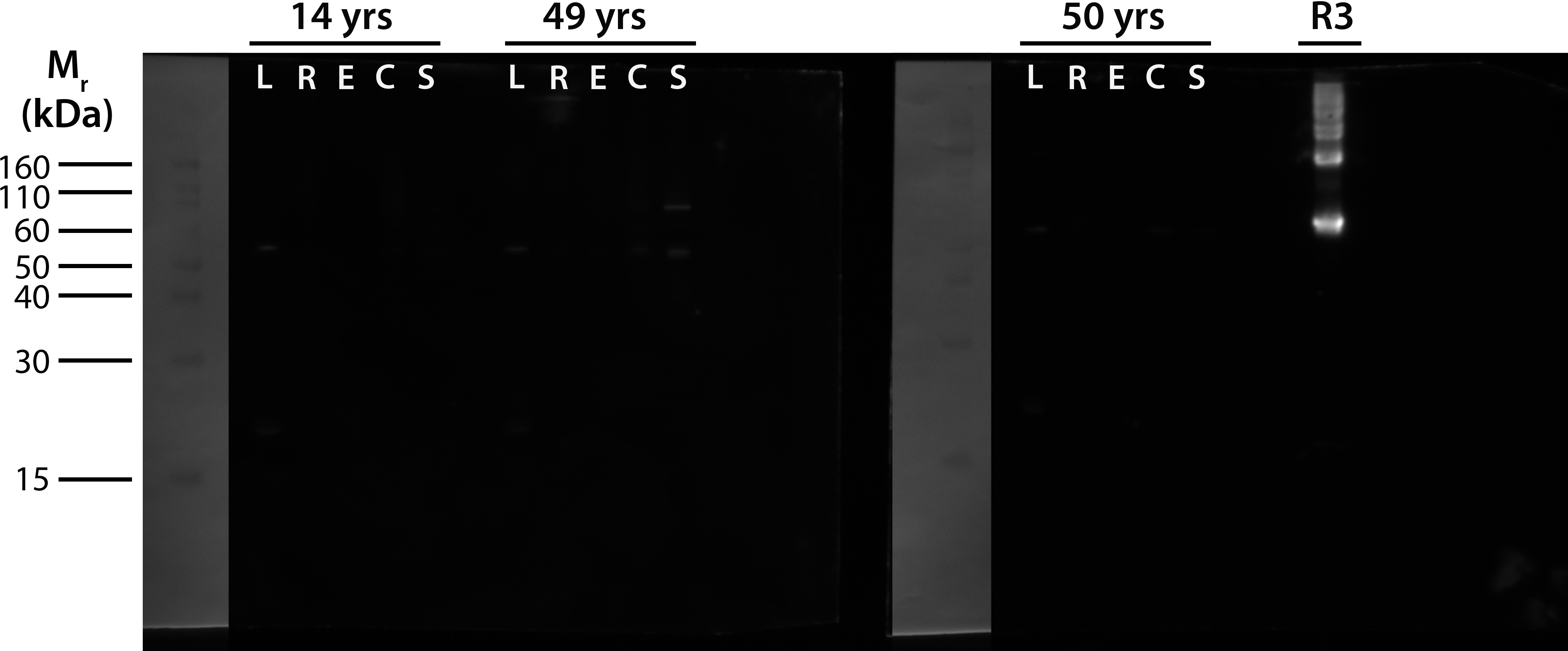

Supplement: S5 Fig — Molecular marker is shown in bright field, while the full western blot is shown as a chemi-luminescent image. L, lens; R, retina; E, RPE; C, choroid; S, sclera. R3, recombinant human RALDH3. Higher molecular weight bands in the “R3” lane are RALDH3 oligomers. (TIF) [file pone.0122008.s005.tif]
